# Di-μ-chlorido-bis­[dichlorido(3,3′,5,5′-tetra­methyl-4,4′-bipyrazol-1-ium-κ*N* ^2′^)copper(II)] dihydrate

**DOI:** 10.1107/S1600536808022605

**Published:** 2008-07-23

**Authors:** Mukhtar A. Kurawa, Christopher J. Adams, A. Guy Orpen

**Affiliations:** a School of Chemistry, University of Bristol, Bristol BS8 1TS, England

## Abstract

The structure of the centrosymmetric title compound, [Cu_2_Cl_6_(C_10_H_15_N_4_)_2_]·2H_2_O, consists of a dimeric [{(HMe_4_bpz)CuCl_3_}_2_] unit (HMe_4_bpz is 3,3′,5,5′-tetra­methyl-4,4′-bipyrazol-1-ium) with two solvent water molecules. Each [HMe_4_bpz]^+^ cation is bonded to a CuCl_3_ unit through a Cu—N dative bond, effectively making square-planar geometry at the Cu atom. Two of these units then undergo a face-to-face dimerization so that the Cu atoms have a Jahn–Teller distorted square-pyramidal geometry with three chlorides and an N atom in the basal plane and one chloride weakly bound in the apical position. Several N—H⋯Cl, O—H⋯Cl and N—H⋯O hydrogen bonds form a three-dimensional network.

## Related literature

We have been unable to find any references in the literature to any other compound containing a monoprotonated 3,3′,5,5′- tetra­methyl­bipyrazole ligand coordinated only to one metal atom through a single nitro­gen donor, but Komarchuk *et al.* (2004 ▶) reported a compound containing two unprotonated tetra­methyl­bipyrazole ligands acting as ligands to a single copper atom. For an exploration of N—H⋯Cl interactions in the design and synthesis of crystal structures with desired properties such as unit-cell metrics or defined reactivity, see: Adams *et al.* (2005 ▶).
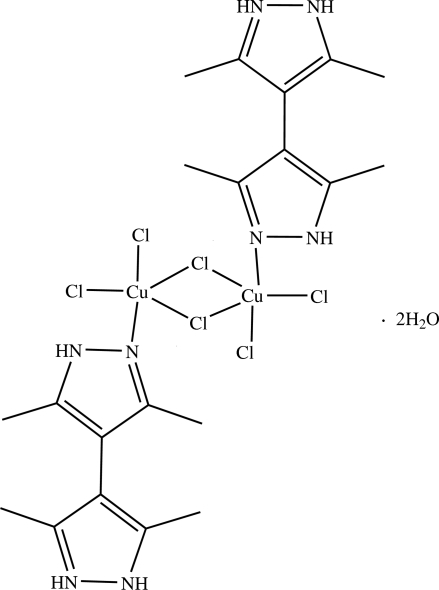


## Experimental

### 

#### Crystal data

[Cu_2_Cl_6_(C_10_H_15_N_4_)_2_]·2H_2_O
*M* *_r_* = 758.35
Triclinic, 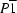

*a* = 8.2837 (4) Å
*b* = 10.5907 (6) Å
*c* = 10.9058 (6) Å
α = 102.4385 (9)°
β = 108.4401 (9)°
γ = 110.2613 (8)°
*V* = 792.70 (7) Å^3^
*Z* = 1
Mo *K*α radiation
μ = 1.88 mm^−1^
*T* = 173 (2) K
0.2 × 0.13 × 0.07 mm

#### Data collection

Bruker SMART CCD area-detector diffractometer
Absorption correction: multi-scan (*SADABS*; Sheldrick, 2008*a* ▶) *T*_min_ = 0.787, *T*_max_ = 0.87
8466 measured reflections
3609 independent reflections
3242 reflections with *I* > 2σ(*I*)
*R*_int_ = 0.023

#### Refinement

*R*[*F* ^2^ > 2σ(*F* ^2^)] = 0.024
*wR*(*F* ^2^) = 0.064
*S* = 1.04
3609 reflections
182 parameters
2 restraints
H atoms treated by a mixture of independent and constrained refinement
Δρ_max_ = 0.39 e Å^−3^
Δρ_min_ = −0.34 e Å^−3^

### 

Data collection: *SMART* (Bruker, 2001 ▶); cell refinement: *SAINT* (Bruker, 2001 ▶); data reduction: *SAINT*; program(s) used to solve structure: *SHELXS97* (Sheldrick, 2008*b* ▶); program(s) used to refine structure: *SHELXL97* (Sheldrick, 2008*b* ▶); molecular graphics: *SHELXTL* (Sheldrick, 2008*b* ▶); software used to prepare material for publication: *SHELXTL*.

## Supplementary Material

Crystal structure: contains datablocks I, global. DOI: 10.1107/S1600536808022605/rn2046sup1.cif

Structure factors: contains datablocks I. DOI: 10.1107/S1600536808022605/rn2046Isup2.hkl

Additional supplementary materials:  crystallographic information; 3D view; checkCIF report

## Figures and Tables

**Table 1:** Selected bond lengths (Å)

Cu1—N2	1.9834 (13)
Cu1—Cl1	2.2684 (5)
Cu1—Cl3	2.2988 (4)
Cu1—Cl2	2.3345 (4)

**Table 2:** Hydrogen-bond geometry (Å, °)

*D*—H⋯*A*	*D*—H	H⋯*A*	*D*⋯*A*	*D*—H⋯*A*
N1—H1*A*⋯Cl1^i^	0.88	2.43	3.2625 (14)	157
N3—H3*A*⋯O1^ii^	0.88	1.80	2.6786 (19)	173
N4—H4*A*⋯Cl3^iii^	0.88	2.26	3.1435 (14)	179
O1—H11⋯Cl1^iv^	0.811 (15)	2.519 (17)	3.2640 (14)	153 (2)
O1—H11⋯Cl3^iv^	0.811 (15)	2.74 (2)	3.3031 (14)	128.5 (19)
O1—H12⋯Cl2^v^	0.800 (15)	2.404 (16)	3.1923 (14)	169 (2)

Symmetry codes: (i) 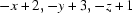; (ii) 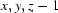; (iii) 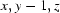; (iv) 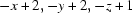; (v) 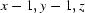.

**Fig. 1.**
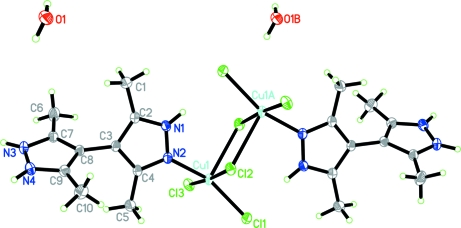
The molecular structure of I with atom labels and 50% probability displacement ellipsoids for non-H atoms. [Symmetry codes: (A) -x + 2, -y + 3, -z + 1; (B) x, y - 1, z]

**Fig. 2.**
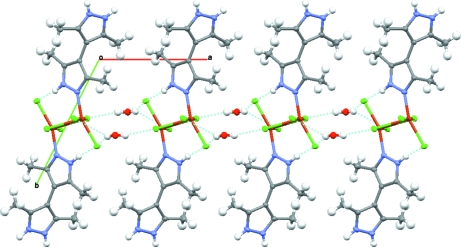
Packing of I viewed down the c axis, with O—H···Cl bonds connecting the dimeric units.

## supplementary crystallographic information

### Comment

We have sought to explore N—H···Cl interactions in designing and synthesizing crystal structures with desired properties such as unit cell metrics or defined reactivity (Adams *et al.*, 2005). We aimed to utilize these interactions by reacting 3,3',5,5'- tetramethylbipyrazole dihydrochloride and copper(II) chloride dihydrate in a 1:1 ratio to synthesize (C_10_H_16_N_4_)[CuCl_4_]. However, the title compound I was obtained instead, crystallizing in the triclinic system with the *P*1 space group, with a [HMe_4_bpz]^+^ cation bonded to a CuCl_3_^- ^unit through a Cu—N bond, forming a zwitterion. In the crystal structure, water molecules bridge adjacent [{(HMe_4_bpz)CuCl_3_}_2_] dimers through O—H···Cl hydrogen bonds forming a hydrogen bonded ribbon (Fig. 2) along the *a*-axis.

### Experimental

An attempt to synthesize tetramethylbipyrazolium tetrachlorocuprate(II) by slow evaporation at room temperature of a solution of equimolar amounts of tetramethylbipyrazole hydrochloride and copper(II) chloride dihydrate in concentrated HCl resulted in the formation of the title compound as a by-product in the form of pale green, plate-like crystals.

### Refinement

H atoms bonded to O atoms were located in a difference map and refined with distance restraints of O—H = 0.84 (2) Å with *U*_iso_(H) = 1.2*U*_eq_(O). Other H atoms were positioned geometrically and refined using a riding model, with C—H = 0.98 Å and N—H = 0.88 Å, with *U*_iso_(H) = 1.2 (1.5 for methyl groups) times *U*_eq_(C, N).

### Crystal data

**Table:**

[Cu_2_Cl_6_(C_10_H_15_N_4_)_2_]·2H_2_O	*Z* = 1
*M**_r_* = 758.35	*F*_000_ = 386
Triclinic, *P*1	*D*_x_ = 1.589 Mg m^−^^3^
*a* = 8.2837 (4) Å	Mo *K*α radiation λ = 0.71073 Å
*b* = 10.5907 (6) Å	Cell parameters from 5647 reflections
*c* = 10.9058 (6) Å	θ = 2.4–27.5º
α = 102.4385 (9)º	µ = 1.88 mm^−^^1^
β = 108.4401 (9)º	*T* = 173 (2) K
γ = 110.2613 (8)º	Plate, green
*V* = 792.70 (7) Å^3^	0.2 × 0.13 × 0.08 mm

### Data collection

**Table:**

Bruker SMART CCD area-detector diffractometer	3609 independent reflections
Radiation source: fine-focus sealed tube	3242 reflections with *I* > 2σ(*I*)
Monochromator: graphite	*R*_int_ = 0.023
*T* = 173(2) K	θ_max_ = 27.5º
φ and ω scans	θ_min_ = 2.2º
Absorption correction: multi-scan(SADABS; Sheldrick, 2008a)	*h* = −10→10
*T*_min_ = 0.787, *T*_max_ = 0.87	*k* = −13→13
8466 measured reflections	*l* = −14→14

### Refinement

**Table:**

Refinement on *F*^2^	Secondary atom site location: difference Fourier map
Least-squares matrix: full	Hydrogen site location: inferred from neighbouring sites
*R*[*F*^2^ > 2σ(*F*^2^)] = 0.024	H atoms treated by a mixture of independent and constrained refinement
*wR*(*F*^2^) = 0.064	*w* = 1/[σ^2^(*F*_o_^2^) + (0.0305*P*)^2^ + 0.3154*P*] where *P* = (*F*_o_^2^ + 2*F*_c_^2^)/3
*S* = 1.04	(Δ/σ)_max_ = 0.002
3609 reflections	Δρ_max_ = 0.39 e Å^−^^3^
182 parameters	Δρ_min_ = −0.33 e Å^−^^3^
2 restraints	Extinction correction: none
Primary atom site location: structure-invariant direct methods	

### Special details

**Table:**

Geometry. All e.s.d.'s (except the e.s.d. in the dihedral angle between two l.s. planes) are estimated using the full covariance matrix. The cell e.s.d.'s are taken into account individually in the estimation of e.s.d.'s in distances, angles and torsion angles; correlations between e.s.d.'s in cell parameters are only used when they are defined by crystal symmetry. An approximate (isotropic) treatment of cell e.s.d.'s is used for estimating e.s.d.'s involving l.s. planes.
Refinement. Refinement of *F*^2^ against ALL reflections. The weighted *R*-factor *wR* and goodness of fit *S* are based on *F*^2^, conventional *R*-factors *R* are based on *F*, with *F* set to zero for negative *F*^2^. The threshold expression of *F*^2^ > σ(*F*^2^) is used only for calculating *R*-factors(gt) *etc*. and is not relevant to the choice of reflections for refinement. *R*-factors based on *F*^2^ are statistically about twice as large as those based on *F*, and *R*- factors based on ALL data will be even larger.

### Fractional atomic coordinates and isotropic or equivalent isotropic displacement parameters (Å^2^)

**Table:**

	*x*	*y*	*z*	*U*_iso_*/*U*_eq_	
Cu1	1.11358 (3)	1.463916 (19)	0.381212 (19)	0.01706 (7)	
Cl1	1.38009 (6)	1.67695 (4)	0.47299 (5)	0.02431 (10)	
Cl2	1.16045 (6)	1.45959 (4)	0.60262 (4)	0.02086 (9)	
Cl3	1.04245 (6)	1.46073 (4)	0.15868 (4)	0.02189 (10)	
N1	0.7878 (2)	1.19314 (14)	0.32487 (14)	0.0185 (3)	
H1A	0.7400	1.2435	0.3637	0.022*	
N2	0.9482 (2)	1.25267 (14)	0.30738 (14)	0.0178 (3)	
N3	0.7030 (2)	0.64464 (15)	0.01465 (15)	0.0210 (3)	
H3A	0.6352	0.5632	−0.0579	0.025*	
N4	0.8615 (2)	0.67473 (15)	0.12509 (15)	0.0217 (3)	
H4A	0.9136	0.6159	0.1356	0.026*	
C1	0.5323 (3)	0.9567 (2)	0.2828 (2)	0.0291 (4)	
H1B	0.5223	1.0103	0.3631	0.044*	
H1C	0.5358	0.8677	0.2923	0.044*	
H1D	0.4220	0.9325	0.1977	0.044*	
C2	0.7099 (2)	1.04760 (17)	0.27573 (17)	0.0185 (3)	
C3	0.8267 (2)	1.01087 (17)	0.22334 (16)	0.0167 (3)	
C4	0.9734 (2)	1.14256 (17)	0.24501 (16)	0.0172 (3)	
C5	1.1377 (3)	1.16715 (19)	0.2077 (2)	0.0261 (4)	
H5A	1.1436	1.2324	0.1559	0.039*	
H5B	1.1210	1.0746	0.1501	0.039*	
H5C	1.2561	1.2106	0.2926	0.039*	
C6	0.4994 (3)	0.7602 (2)	−0.0706 (2)	0.0310 (4)	
H6A	0.4776	0.7067	−0.1642	0.047*	
H6B	0.5248	0.8602	−0.0607	0.047*	
H6C	0.3862	0.7144	−0.0553	0.047*	
C7	0.6656 (2)	0.75913 (17)	0.03346 (17)	0.0192 (3)	
C8	0.8057 (2)	0.86539 (17)	0.16027 (17)	0.0171 (3)	
C9	0.9272 (2)	0.80751 (17)	0.21604 (18)	0.0194 (3)	
C10	1.0961 (3)	0.8699 (2)	0.3517 (2)	0.0290 (4)	
H10A	1.1679	0.8134	0.3499	0.044*	
H10B	1.0542	0.8671	0.4260	0.044*	
H10C	1.1775	0.9703	0.3684	0.044*	
O1	0.50412 (19)	0.40901 (14)	0.78153 (14)	0.0258 (3)	
H11	0.570 (3)	0.402 (2)	0.741 (2)	0.031*	
H12	0.413 (3)	0.410 (2)	0.729 (2)	0.031*	

### Atomic displacement parameters (Å^2^)

**Table:**

	*U*^11^	*U*^22^	*U*^33^	*U*^12^	*U*^13^	*U*^23^
Cu1	0.02091 (11)	0.01281 (11)	0.01527 (11)	0.00629 (8)	0.00744 (8)	0.00371 (8)
Cl1	0.0229 (2)	0.01816 (19)	0.0263 (2)	0.00477 (16)	0.01237 (17)	0.00262 (16)
Cl2	0.0242 (2)	0.0218 (2)	0.0157 (2)	0.01139 (16)	0.00700 (16)	0.00580 (15)
Cl3	0.0302 (2)	0.0242 (2)	0.0190 (2)	0.01682 (18)	0.01283 (17)	0.01042 (16)
N1	0.0212 (7)	0.0162 (7)	0.0193 (7)	0.0090 (6)	0.0104 (6)	0.0050 (5)
N2	0.0208 (7)	0.0148 (6)	0.0168 (7)	0.0072 (6)	0.0085 (6)	0.0045 (5)
N3	0.0246 (7)	0.0150 (7)	0.0197 (7)	0.0082 (6)	0.0085 (6)	0.0026 (6)
N4	0.0265 (7)	0.0186 (7)	0.0239 (8)	0.0138 (6)	0.0112 (6)	0.0080 (6)
C1	0.0264 (9)	0.0215 (9)	0.0384 (11)	0.0074 (7)	0.0192 (8)	0.0069 (8)
C2	0.0202 (8)	0.0166 (8)	0.0171 (8)	0.0085 (7)	0.0068 (6)	0.0051 (6)
C3	0.0180 (7)	0.0152 (7)	0.0144 (8)	0.0074 (6)	0.0049 (6)	0.0042 (6)
C4	0.0204 (8)	0.0157 (7)	0.0145 (8)	0.0087 (6)	0.0061 (6)	0.0047 (6)
C5	0.0269 (9)	0.0202 (8)	0.0331 (10)	0.0099 (7)	0.0172 (8)	0.0075 (7)
C6	0.0270 (9)	0.0258 (9)	0.0267 (10)	0.0127 (8)	−0.0001 (8)	0.0012 (8)
C7	0.0214 (8)	0.0163 (8)	0.0197 (8)	0.0084 (6)	0.0093 (7)	0.0056 (6)
C8	0.0194 (7)	0.0148 (7)	0.0183 (8)	0.0081 (6)	0.0086 (6)	0.0065 (6)
C9	0.0217 (8)	0.0177 (8)	0.0206 (8)	0.0094 (7)	0.0095 (7)	0.0087 (7)
C10	0.0264 (9)	0.0269 (9)	0.0284 (10)	0.0125 (8)	0.0040 (8)	0.0110 (8)
O1	0.0243 (7)	0.0271 (7)	0.0245 (7)	0.0119 (6)	0.0117 (6)	0.0043 (5)

### Geometric parameters (Å, °)

**Table:**

Cu1—N2	1.9834 (13)	C2—C3	1.388 (2)
Cu1—Cl1	2.2684 (5)	C3—C4	1.407 (2)
Cu1—Cl3	2.2988 (4)	C3—C8	1.470 (2)
Cu1—Cl2	2.3345 (4)	C4—C5	1.495 (2)
Cu1—Cl2^i^	2.7029 (5)	C5—H5A	0.9800
Cl2—Cu1^i^	2.7029 (5)	C5—H5B	0.9800
N1—C2	1.348 (2)	C5—H5C	0.9800
N1—N2	1.3538 (19)	C6—C7	1.487 (2)
N1—H1A	0.8800	C6—H6A	0.9800
N2—C4	1.335 (2)	C6—H6B	0.9800
N3—C7	1.342 (2)	C6—H6C	0.9800
N3—N4	1.349 (2)	C7—C8	1.391 (2)
N3—H3A	0.8800	C8—C9	1.400 (2)
N4—C9	1.336 (2)	C9—C10	1.487 (2)
N4—H4A	0.8800	C10—H10A	0.9800
C1—C2	1.490 (2)	C10—H10B	0.9800
C1—H1B	0.9800	C10—H10C	0.9800
C1—H1C	0.9800	O1—H11	0.811 (15)
C1—H1D	0.9800	O1—H12	0.800 (15)
			
N2—Cu1—Cl1	159.99 (4)	C2—C3—C8	127.73 (15)
N2—Cu1—Cl3	90.16 (4)	C4—C3—C8	126.42 (14)
Cl1—Cu1—Cl3	92.769 (17)	N2—C4—C3	109.72 (14)
N2—Cu1—Cl2	87.45 (4)	N2—C4—C5	121.50 (15)
Cl1—Cu1—Cl2	90.762 (17)	C3—C4—C5	128.78 (14)
Cl3—Cu1—Cl2	175.537 (17)	C4—C5—H5A	109.5
N2—Cu1—Cl2^i^	95.31 (4)	C4—C5—H5B	109.5
Cl1—Cu1—Cl2^i^	104.333 (16)	H5A—C5—H5B	109.5
Cl3—Cu1—Cl2^i^	92.434 (14)	C4—C5—H5C	109.5
Cl2—Cu1—Cl2^i^	84.042 (14)	H5A—C5—H5C	109.5
Cu1—Cl2—Cu1^i^	95.958 (14)	H5B—C5—H5C	109.5
C2—N1—N2	111.89 (13)	C7—C6—H6A	109.5
C2—N1—H1A	124.1	C7—C6—H6B	109.5
N2—N1—H1A	124.1	H6A—C6—H6B	109.5
C4—N2—N1	106.26 (13)	C7—C6—H6C	109.5
C4—N2—Cu1	130.17 (11)	H6A—C6—H6C	109.5
N1—N2—Cu1	123.33 (10)	H6B—C6—H6C	109.5
C7—N3—N4	108.88 (13)	N3—C7—C8	107.84 (14)
C7—N3—H3A	125.6	N3—C7—C6	122.10 (15)
N4—N3—H3A	125.6	C8—C7—C6	130.06 (15)
C9—N4—N3	109.65 (13)	C7—C8—C9	106.25 (14)
C9—N4—H4A	125.2	C7—C8—C3	127.55 (14)
N3—N4—H4A	125.2	C9—C8—C3	126.19 (15)
C2—C1—H1B	109.5	N4—C9—C8	107.39 (15)
C2—C1—H1C	109.5	N4—C9—C10	122.58 (15)
H1B—C1—H1C	109.5	C8—C9—C10	130.00 (15)
C2—C1—H1D	109.5	C9—C10—H10A	109.5
H1B—C1—H1D	109.5	C9—C10—H10B	109.5
H1C—C1—H1D	109.5	H10A—C10—H10B	109.5
N1—C2—C3	106.28 (15)	C9—C10—H10C	109.5
N1—C2—C1	122.23 (15)	H10A—C10—H10C	109.5
C3—C2—C1	131.49 (15)	H10B—C10—H10C	109.5
C2—C3—C4	105.84 (14)	H11—O1—H12	107 (2)
			
N2—Cu1—Cl2—Cu1^i^	−95.61 (4)	N1—N2—C4—C5	179.50 (15)
Cl1—Cu1—Cl2—Cu1^i^	104.331 (16)	Cu1—N2—C4—C5	−6.1 (2)
Cl2^i^—Cu1—Cl2—Cu1^i^	0.0	C2—C3—C4—N2	0.31 (18)
C2—N1—N2—C4	0.25 (18)	C8—C3—C4—N2	−178.69 (15)
C2—N1—N2—Cu1	−174.59 (11)	C2—C3—C4—C5	−179.52 (17)
Cl1—Cu1—N2—C4	−27.4 (2)	C8—C3—C4—C5	1.5 (3)
Cl3—Cu1—N2—C4	71.20 (14)	N4—N3—C7—C8	0.06 (19)
Cl2—Cu1—N2—C4	−112.57 (14)	N4—N3—C7—C6	179.90 (16)
Cl2^i^—Cu1—N2—C4	163.66 (14)	N3—C7—C8—C9	−0.39 (19)
Cl1—Cu1—N2—N1	146.16 (10)	C6—C7—C8—C9	179.78 (18)
Cl3—Cu1—N2—N1	−115.29 (12)	N3—C7—C8—C3	178.39 (16)
Cl2—Cu1—N2—N1	60.94 (12)	C6—C7—C8—C3	−1.4 (3)
Cl2^i^—Cu1—N2—N1	−22.83 (12)	C2—C3—C8—C7	69.0 (3)
C7—N3—N4—C9	0.32 (19)	C4—C3—C8—C7	−112.3 (2)
N2—N1—C2—C3	−0.06 (18)	C2—C3—C8—C9	−112.5 (2)
N2—N1—C2—C1	179.98 (15)	C4—C3—C8—C9	66.3 (2)
N1—C2—C3—C4	−0.15 (18)	N3—N4—C9—C8	−0.56 (19)
C1—C2—C3—C4	179.81 (18)	N3—N4—C9—C10	177.32 (16)
N1—C2—C3—C8	178.83 (15)	C7—C8—C9—N4	0.58 (19)
C1—C2—C3—C8	−1.2 (3)	C3—C8—C9—N4	−178.22 (16)
N1—N2—C4—C3	−0.34 (17)	C7—C8—C9—C10	−177.09 (18)
Cu1—N2—C4—C3	174.02 (11)	C3—C8—C9—C10	4.1 (3)

Symmetry codes: (i) −*x*+2, −*y*+3, −*z*+1.

### Hydrogen-bond geometry (Å, °)

**Table:**

*D*—H···*A*	*D*—H	H···*A*	*D*···*A*	*D*—H···*A*
N1—H1A···Cl1^i^	0.88	2.43	3.2625 (14)	157
N3—H3A···O1^ii^	0.88	1.80	2.6786 (19)	173
N4—H4A···Cl3^iii^	0.88	2.26	3.1435 (14)	179
O1—H11···Cl1^iv^	0.811 (15)	2.519 (17)	3.2640 (14)	153 (2)
O1—H11···Cl3^iv^	0.811 (15)	2.74 (2)	3.3031 (14)	128.5 (19)
O1—H12···Cl2^v^	0.800 (15)	2.404 (16)	3.1923 (14)	169 (2)

Symmetry codes: (i) −*x*+2, −*y*+3, −*z*+1; (ii) *x*, *y*, *z*−1; (iii) *x*, *y*−1, *z*; (iv) −*x*+2, −*y*+2, −*z*+1; (v) *x*−1, *y*−1, *z*.
